# Correction: Quan et al. Functional Characterization of a Novel SMR-Type Efflux Pump RanQ, Mediating Quaternary Ammonium Compound Resistance in *Riemerella anatipestifer*. *Microorganisms* 2023, *11*, 907

**DOI:** 10.3390/microorganisms13051143

**Published:** 2025-05-16

**Authors:** Heng Quan, Xiaowei Gong, Wenhui Wang, Fuying Zheng, Yongfeng Yu, Donghui Liu, Qiwei Chen, Yuefeng Chu

**Affiliations:** 1 College of Veterinary Medicine, Gansu Agricultural University, Lanzhou 730070, China; quanheng0517@163.com (H.Q.); donghuiliu63@163.com (D.L.); 2 State Key Laboratory of Veterinary Etiological Biology, College of Veterinary Medicine, Lanzhou University, Lanzhou Veterinary Research Institute, Chinese Academy of Agricultural Sciences, Lanzhou 730000, China; gongxiaowei@caas.cn (X.G.); zhengfuying@caas.cn (F.Z.); yuyongfengvip@163.com (Y.Y.); chuyuefeng@caas.cn (Y.C.)

In the original publication [1], the order of the authors was incorrect. The corrected order appears below.

Heng Quan ^1,2^, Xiaowei Gong ^2^, Wenhui Wang ^1,^*, Fuying Zheng ^2^, Yongfeng Yu ^2^, Donghui Liu ^1,2^, Qiwei Chen ^2^,* and Yuefeng Chu ^2^

The authors state that the scientific conclusions are unaffected. This correction was approved by the Academic Editor. The original publication has also been updated.
